# Supplementary figures and images for: Isolation and Characterization of Streptococcus mutans Phage as a Possible Treatment Agent for Caries
Source: Viruses. 2021 May 2;13(5):825. doi: 10.3390/v13050825 (PMC8147482; doi:10.3390/v13050825)

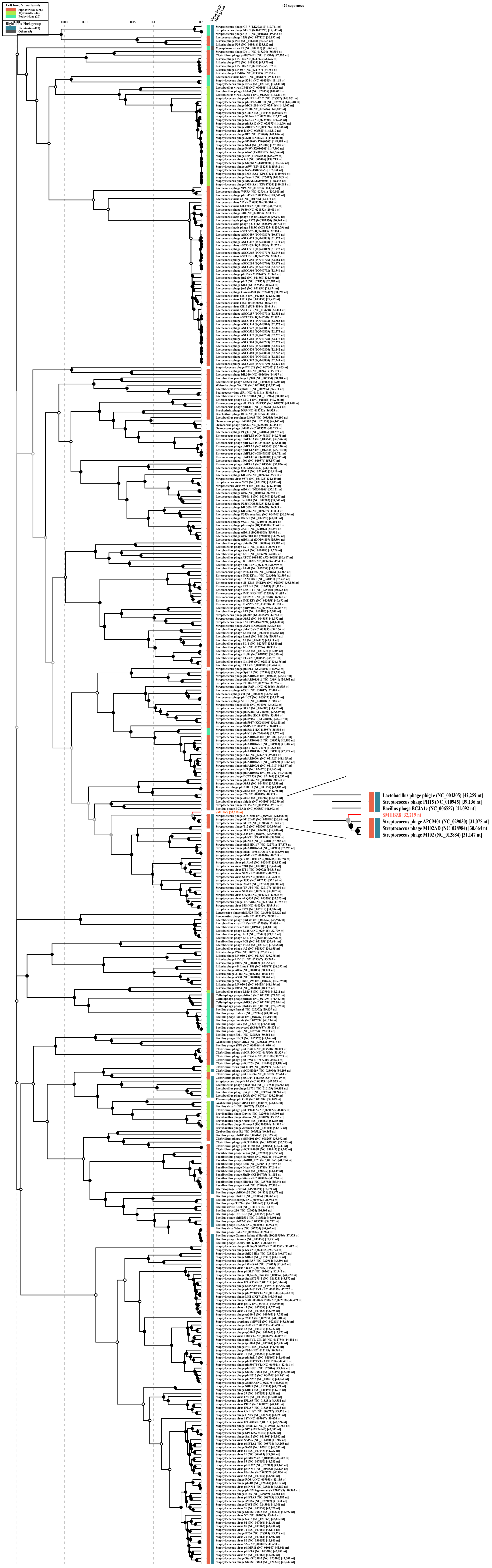

Supplement: Supplementary file 1 [file viruses-13-00825-s001.zip › Figure S1 VipTree.pdf]
